# Patients’ and clinicians’ perspectives on a ‘fast-track’ pathway for patients with sciatica in primary care: qualitative findings from the SCOPiC stratified care trial

**DOI:** 10.1186/s12891-020-03483-z

**Published:** 2020-07-17

**Authors:** Benjamin Saunders, Kika Konstantinou, Majid Artus, Nadine E Foster, Bernadette Bartlam

**Affiliations:** 1 Primary Care Centre Versus Arthritis, School of Primary, Community and Social Care, Keele University, Staffordshire, ST5 5BG UK; 2 Haywood Hospital, Midlands Partnership Foundation NHS Trust, Staffordshire, UK; 3 Keele Clinical Trials Unit (CTU), David Weatherall Building, Keele University, Staffordshire, ST5 5BG UK

**Keywords:** Sciatica, Stratified care, ‘Fast-track’ pathway, Qualitative, Interviews

## Abstract

**Background:**

Sciatica is common and associated with significant impacts for the individual and society. The SCOPiC randomised controlled trial (RCT) (trial registration: ISRCTN75449581) tested stratified primary care for sciatica by subgrouping patients into one of three groups based on prognostic and clinical indicators. Patients in one group were ‘fast-tracked’ for a magnetic resonance imaging (MRI) scan and spinal specialist opinion. This paper reports qualitative research exploring patients’ and clinicians’ perspectives on the acceptability of this ‘fast-track’ pathway.

**Methods:**

Semi-structured interviews were conducted with 20 patients and 20 clinicians (general practitioners, spinal specialist physiotherapists, spinal surgeons). Data were analysed thematically and findings explored using Normalisation Process Theory (NPT) and ‘boundary objects’ concept.

**Results:**

Whilst the ‘fast-track’ pathway achieved a degree of ‘coherence’ (i.e. made sense) to both patients and clinicians, particularly in relation to providing early reassurance based on MRI scan findings, it was less ‘meaningful’ to some clinicians for managing patients with acute symptoms, reflecting a reluctance to move away from the usual ‘stepped care’ approach. Both groups felt a key limitation of the pathway was that it did not shorten patient waiting times between their spinal specialist consultation and further treatments.

**Conclusion:**

Findings contribute new knowledge about patients’ and clinicians’ perspectives on the role of imaging and spinal specialist opinion in the management of sciatica, and provide important insights for understanding the ‘fast-track’ pathway, as part of the stratified care model tested in the RCT.

Future research into the early referral of patients with sciatica for investigation and specialist opinion should include strategies to support clinician behaviour change; as well as take into account the role of imaging in providing reassurance to patients with severe symptoms in cases where imaging reveals a clear explanation for the patient’s pain, and where this is accompanied by a thorough explanation from a trusted clinical expert.

## Background

Sciatica is a common form of low back pain (LBP) characterised by pain radiating into the leg [1]. Unlike LBP alone, sciatica often has a clear ‘biomedical’ cause, most commonly a disc prolapse compressing or irritating a spinal nerve root [2]. Sciatica is often experienced as acute symptoms resolving over several weeks or months, either naturally or with treatment; however, up to 30% of people will still experience pain after a year [1]. When compared to LBP alone, patients with sciatica have worse pain and disability, poorer quality of life and use more healthcare resources [3, 4].

Current usual clinical management for most patients with sciatica follows a ‘stepped’ care approach, with initial conservative management in primary care comprising low-intensity treatments (e.g. advice and education, ‘wait-and-see’ approaches, pain medications), before moving to more intensive treatments (e.g. courses of physiotherapy) if symptoms persist. Patients whose symptoms still fail to improve are referred to specialist spinal services for investigations and consideration for more invasive treatments such as spinal injections and surgery.

An alternative to stepped care is a stratified care approach, whereby subgroups of patients are matched to appropriate treatments early on. Stratified care aims to ‘identify those who will have the most clinical benefit or least harm from a specific treatment’ in order to ‘make the best decisions for groups of similar patients’ [5]. In non-specific LBP, a model of stratified care based on prognostic risk of persistent disabling back pain, using a brief self-report tool – the STarT Back tool [6] and matched treatments, has been shown to be superior to non-stratified care both in terms of clinical outcomes and cost-effectiveness [7, 8].

Building on this evidence, the SCOPiC (SCiatica Outcomes in Primary Care) randomised controlled trial (RCT) (Trial registration: ISRCTN75449581), tested the clinical and cost-effectiveness of a new model of stratified care for UK National Health Service (NHS) sciatica patients consulting in primary care, compared with usual, non-stratified care. Eligible patients randomised to the intervention arm were stratified into one of three groups based on a combination of prognostic and clinical information [9]. Each of the groups was matched to a care pathway. Patients in group 1 received brief self-management support (up to 2 sessions with a physiotherapist). Those in group 2 received a course of up to 6 sessions of physiotherapist-led care. Patients in group 3 were ‘fast-tracked’ for a Magnetic Resonance Imagining scan (MRI) and spinal specialist consultation within 4 weeks, for consideration of their suitability for other more invasive treatments, such as spinal injections or surgery. After that point, patient care and any onward treatments such as injections or surgery followed routine NHS practice and waiting times. The trial showed that this stratified care model was not superior to usual, non-stratified care for the primary outcome: time to first symptom resolution [10]. The majority of participants in both of the arms of the trial reported resolution over the 12 months of the trial.

This paper reports on the findings from nested qualitative research that aimed to understand the acceptability of the ‘fast-track’ pathway to patients and clinicians, i.e. general practitioners (GPs), spinal specialist physiotherapists and spinal surgeons. The reason for focusing on this aspect of the trial specifically is the novelty of the ‘fast-track’ pathway to MRI and spinal specialist opinion. The development of the ‘fast-track’ pathway was based on evidence of variation in clinical practice in the UK NHS in terms of referrals from general practice to specialist spinal services, resulting in delays in some patients with severe pain being referred to specialist services, and subsequently delays in these patients receiving appropriate treatments [11]. As a result, the UK Spinal Taskforce [11] identified the need for evidence on the clinical and cost effectiveness of early referral of patients with severe symptoms for consideration of treatments such as surgery or spinal epidural injections, to assess whether ‘fast-tracking’ a group of patients can lead to improvement in these patients’ outcomes. See [10] full details of the trial procedures.

However, clinicians’ and patients’ views towards the acceptability of ‘fast-tracking’ patients with severe sciatica symptoms for early investigation and spinal specialist opinion, and how this is experienced in practice, is unknown. This is important, as exploring how a care pathway is perceived and experienced by patients and clinicians in everyday practice can provide important context for interpreting outcomes in pragmatic RCTs [12, 13]. The findings presented in this paper provide useful insights for understanding this element of the stratified care model that was tested, and whilst, based on the RCT findings there is no strong justification to implement this model of stratified care, other models of stratification could be developed and would require testing. The insights from the qualitative findings can therefore have potential implications for future intervention design, as well as future clinical management of patients with sciatica.

## Theoretical frameworks

In the absence of suspected serious pathology, the ‘fast-track’ care pathway clearly represented a change to usual care management for a subgroup of patients with sciatica. Changing healthcare practice holds diverse challenges [14] depending on whether an intervention is considered meaningful and relevant to clinicians and patients, and the degree to which the intervention is seen to ‘fit’ within established ways of working [15]. Examining such issues using a theoretically-underpinned approach can extend the scope of purely descriptive approaches, enabling a more cogent and coherent explanation of the issues identified in the data. As such, we drew on two theoretical frameworks to guide the inquiry. The first was Normalisation Process Theory (NPT) [16], a theoretical framework that has been widely used to explore the introduction of new healthcare interventions across a diverse range of settings, e.g. low back pain [17]; cancer care [18]; and mental health [19]. NPT provides a framework for understanding why some healthcare interventions are accepted and more successfully embedded in routine practice than others [20]. It aims to identify and explain ‘factors that promote and inhibit the routine incorporation of complex interventions into everyday practice’ [20], through highlighting the ‘ways in which work must be reconfigured both individually and collectively by multiple stakeholders involved in the work of implementation’ [21], thus taking into account both the individual level and broader system and organisation level factors. This aim is reflected in the framework’s four main components, as outlined in Murray et al. [20]: coherence (or sense-making); cognitive participation (or engagement); collective action (work done to enable the intervention to happen); and reflexive monitoring (formal and informal appraisal of the benefits and costs of the intervention). We drew on the concept of ‘coherence’ in particular as a lens through which to interpret our interview findings. Coherence relates to the degree to which a new intervention is seen to be ‘meaningful’ to key stakeholders, and whether it ‘makes sense’ within the context of existing ways of working. Whether clinicians and patients ‘buy in’ to a new approach is key to its early adoption and implementation. The concept of coherence thus aligns closely with our aims, as the degree to which clinicians and patients perceive the ‘fast-track’ pathway as making sense in the context of the existing care pathway for sciatica is key in understanding its overall acceptability.

The second theoretical framework used was Allen’s [22] conceptualisation of care pathways as ‘boundary objects’. Allen defines a ‘boundary object’ as a loose concept, but with strong cohesive power, which enables the bringing together of the interests of different groups, whilst still allowing these groups to maintain their respective social identities. Allen argues that care pathways can be considered as ‘boundary objects’ in that they have the ability to ‘align clinical, management and service user interests around a healthcare quality agenda.’ [22:355]. She also points out, however, that they can at the same time give rise to conflicting agendas among different stakeholders (e.g. clinicians, patients, managers) which results in challenges when trying to meet the needs of those groups. This ‘boundary object’ conceptualisation was also drawn-upon as a lens through which to interpret our findings, enabling the investigation of how the ‘fast-track’ pathway addressed the shared goals of patients and the three clinician groups (GPs, spinal specialist physiotherapists and spinal surgeons), aspects in which the ‘fast-track’ pathway did not align with the priorities of these respective groups, and where competing agendas were present in terms of its goals, components and operationalisation.

## Methods

One-to-one semi-structured interviews were conducted with patients on the ‘fast-track’ pathway in the stratified care arm of the SCOPiC trial (*n =* 20), and clinicians (*n =* 20; 7 spinal physiotherapists; 9 GPs; 4 spinal surgeons) between January 2016 and February 2018. Interviews were carried out by the lead author, BS (male, PhD), a social science researcher with significant qualitative research experience. The three clinician groups had differing levels of involvement in the trial (the published trial protocol paper provides full details [23]). GPs identified patients with suspected sciatica for invitation to the SCOPiC research clinics for assessment of their eligibility to participate in the trial. Spinal specialist physiotherapists were directly involved in providing spinal specialist opinion as part of the ‘fast-track’ pathway in NHS spinal interface clinics – these are multi-professional services at the interface between primary and secondary care in the NHS. Spinal surgeons were not involved in assessing patients as part of the ‘fast-track’ pathway following their MRI scan. Some patients were referred from the interface clinics for a consultation with a surgeon, however the surgeons would not necessarily be aware that a patient was participating in the trial.

The study settings were general practices, NHS based spinal clinics and secondary care NHS Trusts in the Midlands of England, North West England and Wales, UK. The study received ethical approval from the NRES Committee West Midlands – Solihull, 17/03/2015, ref.: 15/WM/0078.

### Recruitment

Patients were recruited by invitation letter and then via phone, having consented to contact as part of their participation in the RCT. Patients were purposively sampled to capture diverse characteristics including treatment centre attended, and participant demographics: age, gender, leg pain intensity, treatments received (including patients who had received invasive treatments such as spinal injections or surgery) and response to treatment. Clinicians were initially approached via email (in the case of some GPs via their practice manager), followed by a telephone call or email. Clinicians were sampled for variation in geographical location across the areas involved in the trial (Staffordshire, North Shropshire, Cheshire, and Wales). GPs were further purposively sampled based on the number of patients during the trial for whom a sciatica related Read code (i.e. symptom/diagnostic code) had been entered, prompting a study specific electronic ‘pop-up’ on the GP’s computer system indicating that the patient may be eligible for invitation to the SCOPiC clinics. It was felt that interviewing those GPs who had had most opportunity to engage with the trial in terms of identifying patients to be invited to the SCOPiC research clinics, might yield rich interview data. Sampling based on level of engagement was not relevant for the other clinician groups; the spinal physiotherapists had, by definition, been involved in the management of this group of patients in terms of providing spinal specialist consultations as part of the ‘fast-track’ pathway; spinal surgeons would not have been aware of which patients they had seen that were part of the ‘fast-track’ pathway (see [23] for a full details of the trial procedures). Out of 32 patients invited to interview, 12 patients declined to take part, citing lack of time to participate. No clinicians declined to participate, and no patients or clinicians withdrew once having agreed to be interviewed.

### Data collection

Interviews with patients, spinal physiotherapists and spinal surgeons were conducted after the 4-month follow-up point in the RCT. This was to allow patients to reflect upon their experiences of the ‘fast-track’ pathway up to that point, and for clinicians to have had the opportunity to see patients as part of the ‘fast-track’ pathway. Interviews with GPs were conducted once recruitment to the trial had finished (November 2017 onwards) to avoid the possibility of influencing their normal referral patterns of patients with sciatica. Of the 20 patient interviews, 13 took place at participants’ homes; two at the University; and five via telephone, in line with participants’ preferences. Interviews lasted between 21 min and 1 h 15 min (average: 48 min). Of the 20 clinician interviews, eight were carried out at the clinician’s practice or hospital; four at the University and eight via telephone. Clinician interviews lasted between 19 min and 32 min (average: 26 min).

Interviews were audio-recorded, with the exception of one GP interview in which the GP consented to take part in an interview but not to being audio-recorded. Instead, the interviewer took detailed notes during and immediately after the interview to capture that GP’s views. All participants were given an information letter explaining the study prior to providing written informed consent at the start of interviews, or audio-recorded consent in the case of telephone interviews. Consent was reaffirmed verbally at the end of each interview.

Separate topic guides were used for patient and clinician interviews, covering a range of areas relevant to the qualitative study aims (see Appendix). However, the interviewer still retained flexibility to follow up on any unexpected findings emerging during the interview. Early findings informed subsequent interviews, with the topic guides iteratively revised throughout the data-collection process. Field notes were not made during interviews as it was felt this could negatively impact upon the rapport between interviewer and interviewee.

### Analysis

Audio-recordings of interviews were transcribed and anonymised. A two stage analysis framework was adopted incorporating an inductive thematic analysis [24] followed by mapping the identified themes onto the two theoretical frameworks: the ‘coherence’ construct within NPT and the conceptualisation of care pathways as ‘boundary objects’. Analysis was an iterative process and data collection continued until saturation was judged to have been reached, defined as ‘informational redundancy’– the point at which additional data no longer offers new insights [25].

Anonymised transcripts were first systematically coded on a line-by-line basis by one of the authors (BS) with the aid of the software program Nvivo 10, in order to identify recurrent concepts inductively. Coding was at first largely descriptive, and later became more conceptual as interpretations of the data moved towards a higher level of theoretical abstraction. Coding was reflexive and recursive, with codes being revisited in light of the findings of subsequent data-collection. A random sample of 6 patient transcripts and 6 clinician transcripts was independently coded by three other members of the team. Coders brought different disciplinary perspectives to the data (BS medical sociology; BB social science; MA clinical academic general practice; KK clinical academic physiotherapy and spinal specialist expertise). The aim was to understand cross-disciplinary perspectives on the data and, through discussion, to come to an agreement on shared meanings and interpretations.

Member-checking – in terms of sending transcripts and findings to participants for comment and feedback ─ was not employed. This was principally due to the across-case, rather than within-case, focus of the analysis, which meant it would have been difficult for participants to comment on the validity of the interpretations of their own data when included within an analysis of the broader dataset. Whilst we chose not to seek participants’ feedback on the across-case findings, we do acknowledge this could have offered some additional insights on the findings. Instead, participants were given the opportunity to receive a copy of the findings upon completion of the analysis. Patient perspectives were included in the data analysis, however, as early findings from the patient interview data, along with a sample of three interview transcripts, were shared with four patients from the SCOPiC trial’s Patient and Public Involvement and Engagement (PPIE) group. A meeting was held in which the researchers looked through these transcripts with the PPIE members and explored whether their interpretations of the data aligned with the emergent findings. All four PPIE members were broadly in agreement with the early interpretations of the interview data. Following full analysis, findings were again presented to the same PPIE group. All four PPIE members expressed agreement with the final interpretations of the data and finalised themes. The researchers also discussed with PPIE members the implications of the findings, gaining their views on how findings might inform future research and clinical practice. The implications of the findings will be outlined later in the Discussion and Conclusion sections.

Data were analysed thematically using the constant comparison method [26], looking for connections within and across interviews, and across codes, highlighting data consistencies and variations. Whilst patient and clinician data were initially coded separately, they were then mapped onto one another, looking at how each of the main themes identified did or did not manifest across both the clinician and patient interviews. The second stage of the analysis involved mapping the themes identified in both the patients’ and clinicians’ interviews onto the ‘coherence’ construct of NPT and the conceptualisation of care pathways as ‘boundary objects’. We explored the degree to which the identified themes could be seen to ‘fit’ within these frameworks, and how the theoretical constructs manifested in relation to these themes. In what follows we outline the characteristics of the participant sample, before reporting the key themes.

## Results

### Patient participant characteristics

Ten patient participants were female and 10 male, aged from 28 to 86 years (average age: 52), and represented a range of occupation types. Leg pain intensity measured at the 4-month follow-up point in the RCT varied widely between 1/10 and 10/10, with an average of 5.3/10. Symptom duration for the current episode of sciatica ranged from symptoms having resolved by 3 months, to symptoms still experienced at 11–16 months (average: 5–6 month symptom duration). Table 1, below, summarises the characteristics of the 20 patients interviewed:

**Table 1:** Summary of Patient Participant Characteristics: Provides a summary of the characteristics of the 20 patients interviewed

Patient ID	Age	Gender	Self-reported occupation type	Duration of current symptoms in months at time of interview (4-month follow-up)	Leg pain intensity over past 2 weeks (at 4-month follow-up)	Self-reported symptoms at 4-month follow-up compared to baseline
				*For five patients, symptoms had resolved prior to being interviewed, as indicated below		
1	62	F	Unemployed due to sciatica	5–6	9/10	Worse
2	36	F	Radiographer	Symptoms resolved prior to interview, following 3 month duration	0/10	Completely recovered
3	53	M	Compliance director	7–10	4/10	Better
4	49	F	Pottery worker	7–10	7/10	Same
5	44	M	Assistant manager	9–12	7/10	Same
6	60	F	Dining hall assistant	Symptoms resolved prior to interview, following 3 month duration	3/10	Better
7	64	M	Retired	7–10	9/10	Much worse
8	42	M	Builder	9–12	10/10	Much worse
9	44	F	Early years practitioner	7–10	7/10	Same
10	57	M	Pottery worker	5–6	4/10	Better
11	66	M	Ambulance driver	6–8	3/10	Better
12	86	F	Retired	Symptoms resolved prior to interview, following 4 months’ duration	2/10	Much better
13	45	F	Data claims manager	5–6	7/10	Better
14	69	F	Retired	11–16	6/10	Better
15	55	M	Machine driver	5–6	2/10	Better
16	67	F	Unpaid carer	7–10	7/10	Same
17	70	F	Retired	5–6	6/10	Better
18	67	M	Office manager	Symptoms resolved prior to interview, following 4 month duration	2/10	Much better
19	46	M	Police service staff	5–6	5/10	Better
20	28	M	Left skilled labour job due to sciatica	Symptoms resolved prior to interview, following 5 month duration	1/10	Completely recovered

**Fig. 1:**
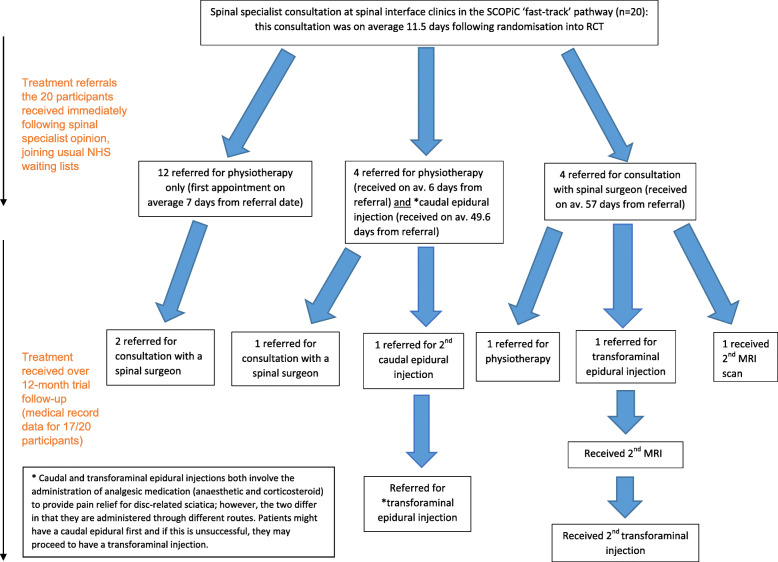
Information on referrals, investigations and treatments received by patient interview participants during the trial: Displays information on investigations, referrals and treatments received by the patients participating in the interviews, along with average waiting times for receipt of treatments from the time of referral

Information was available from clinical report forms about the care patients received following their spinal specialist consultation for all 20 participants. Additional medical record data about care was then retrieved after the 12 months of the total RCT follow-up, and these data were available for 17 of the 20 participants. Since patient interviews were conducted after the 4-month RCT follow-up point, some medical record data postdate the interviews. Figure 1, below, displays information on investigations, referrals and treatments received by the patients participating in the interviews, along with average waiting times for receipt of treatments from the time of referral. Additionally, whilst 4 patients reported in interviews that they had either undergone or were awaiting spinal surgery, this information was not available in the medical record data.

### Clinician participant characteristics

Eleven clinicians were female and nine male. Of these, 5 GPs were female and 4 male, 6 spinal physiotherapists were female and 1 male, and all 4 spinal surgeons were male. The length of time clinicians had been practising ranged from 9 to 31 years. GPs were reasonably equally spread across the geographical regions involved in the trial.

### Principal findings

Three main themes were identified in both the patient and clinician datasets; however, in relation to the patient interviews one theme was not identified in the clinician data: ‘impact on life and sense of identity’. Though clinicians expressed an awareness of the impact of sciatica on patients’ lives, they discussed this issue mainly in relation to how it informed their clinical management. Whilst the other three themes relate directly to the experiences and perceptions of the ‘fast-track’ pathway, this theme as it emerged in the patient data is broader in scope and focused primarily on experiences outside of the healthcare setting. For this reason, this theme has been reported elsewhere [27]. The three key themes identified in relation to both the patient and clinician data were:

1. Acceptability of the ‘fast-track’ care pathway
2. Perceived benefits of the ‘fast-track’ care pathway
3. Waiting times for onward treatment following being ‘fast-tracked’

A summary of the findings in relation to each of these key themes is presented in Table 2, below:

**Table 2:** Summary of key themes: Provides a summary of the findings in relation to each of the key themes identified from the data

Theme	Findings summary
Acceptability of the ‘fast-track’ care pathway	Both patients and clinicians found it acceptable for patients identified as needing spinal specialist assessment and consideration for more invasive treatments to be seen by those specialists sooner. Patients were pleased with the speed of their ‘fast-track’ referral; however, some clinicians expressed concern that patients with short symptom duration may be ‘fast-tracked’ too soon and that their symptoms could still resolve naturally. All clinicians expressed reluctance to consider invasive treatment options too early for these ‘acute’ patients.
Perceived benefits of the ‘fast-track’ care pathway	Patients and clinicians perceived benefits from the ‘fast-track’ pathway in providing early patient reassurance based on MRI scan findings, particularly in enabling patients to understand the cause of their pain and assuring them that there was no serious underlying pathology. However, clinician views were mixed about the potential longer-term clinical benefits of the ‘fast-track’ care pathway.
Waiting times for onward treatment following being ‘fast-tracked’	Although patient management following ‘fast-track’ was not altered as part of the trial, patients highlighted the significant difference between the short timeframe for the initial ‘fast-track’ to MRI scan and spinal specialist opinion and the usual NHS waiting times after onward referral for more invasive treatments. This led to uncertainty about how long they would be waiting to receive further treatments, resulting in dissatisfaction. Clinicians similarly felt that not being able to influence the timing of the receipt of further treatments once patients joined the waiting lists was a limitation of the ‘fast-track’ pathway.

### Acceptability of the ‘fast-track’ care pathway

All patients reported being pleased and surprised by the speed of receiving an MRI scan (which was on average 5 days following randomisation in the RCT) and subsequent spinal specialist appointment at an interface clinic (average: 11.5 days following randomisation). Patients drew contrast between this and their previous experiences of long NHS waiting times for investigations and treatments:

#### Extract 1

*Patient ID 4: I went for my scan more or less straight after [the SCOPiC clinic appointment]. I even commented how quick it was, because normally you wait and wait and wait, don’t you? I was shocked and pleased because … It seemed like I’d jumped the queue! (Female, aged 49)*

Clinicians reported finding it acceptable for patients identified as needing spinal specialist assessment and consideration for more invasive treatments to be seen by those specialists sooner than is usually the case in current practice. In particular, clinicians expressed positive views that for those patients who were suitable for invasive treatments, ‘fast-tracking’ them through the initial phase of the care pathway and onto NHS waiting lists sooner reduced the overall time period it took for them to receive these treatments, enabling the patient’s pain to resolve more quickly and allowing them to get back to work and usual activities sooner:

#### Extract 2

*Spinal physiotherapist 2: There was a patient I saw six or seven weeks down the line, and they were struggling. They were quite severe and their scan findings said they had a big disc problem and they went on to have a caudal epidural injection. They got that injection a lot quicker than they would probably for the normal route, and that helped them massively in the sense that they’ve got back to work.*

However, there was some variation across clinicians in their views about the timing of the ‘fast-track’ pathway based on symptom duration. Thirteen clinicians (7 GPs, 6 spinal physiotherapists) expressed concern that some patients with short symptom duration (i.e. under 6 weeks) may be ‘fast-tracked’ too soon and that their symptoms could still resolve naturally without the need for investigation:

#### Extract 3

*GP 7: My cut-off is if things are no better after six to eight weeks then it’s looking more like a chronic issue that’s maybe not going to resolve easily or quickly. So I would say patients are being fast-tracked for a scan earlier than I would [normally]. Potentially you may be seeing people that would have gone on to just resolve in another two or three weeks.*

These views were reflected in examples given by spinal physiotherapists of patients seen as part of the ‘fast-track’ pathway. Whilst the spinal physiotherapists felt that most patients were ‘fast-tracked’ appropriately, they reported seeing some patients with symptoms of only a few weeks’ duration, and based on their assessment they felt that these patients did not need to see a spinal specialist, particularly if they were beginning to show signs of improvement:

#### Extract 4

*Spinal physiotherapist 5: If you had a DVD (i.e. video) that went through everything, then that would have been just as good as me being there. As long as they were getting better at that point, I don’t really feel they needed to be seen by me really, in terms of a spinal specialist opinion.*

In line with the views in Extracts 3 and 4, above, several clinicians felt that ‘fast-tracking’ may be more beneficial for patients who have had symptoms beyond a certain duration, e.g. 6–10 weeks:

#### Extract 5

*Spinal physiotherapist 7: The only place where it [the ‘fast-track’ pathway] maybe falls down is them being referred into that service too soon. I saw a lady who had only had a two-week history of symptoms and for me, that’s too early to refer into that kind of service. But the ones that are a little bit later down the line, then it’s great to be able to assess them and have all the investigations done relatively rapidly because then you can make a decision quickly for that patient. It’s just where the starting point begins, and making sure the patients don’t come into it too early … I’d say anyone that’s coming through [for spinal specialist opinion] with less than two months or even up to ten weeks’ worth of symptoms, I’d like them to have longer to settle conservatively beforehand.*

However, in contrast, 7 clinicians – all 4 surgeons, 1 spinal physiotherapist and 2 GPs – did see it as acceptable for patients with severe symptoms even if of short duration to be ‘fast-tracked’ as early as possible to a spinal specialist (i.e. even a few weeks since symptom onset). However, this was primarily for the purpose of providing patient reassurance rather than to inform treatment decisions. The concept of reassurance is discussed in greater detail later under the third theme: Perceived benefits of the ‘fast-track’ care pathway.

#### Extract 6

*Spinal surgeon 4: I don’t think getting MRIs done earlier would be a bad idea. Patients like to have that reassurance. So I think the MRI scan is more of a tool to address their mental state rather than to decide treatment from that point of view … I don’t think it will change my surgical plan or decision making or time to treat.*

Whilst there was variation amongst clinicians as to the acceptability of the ‘fast-track’ pathway for patients with short symptom durations, there was greater agreement across the three clinician groups about the management of these ‘acute’ patients following ‘fast-track’ for MRI scan and spinal specialist opinion at the spinal interface clinics. Clinicians expressed reluctance to consider invasive treatment options too early for these ‘acute’ patients, particularly until conservative treatment had first been tried and failed. There was clearly a reticence among clinicians to move away from their usual stepped care model for sciatica patients with short symptom durations:

#### Extract 7

*Spinal surgeon 1: Even if I see patients privately, I tell them to wait for two to three months anyway; even if they are paying me I will say wait two or three months so that is our standard approach, that's what we follow.*

*Interviewer: So if a patient did come to you earlier would you still take that conservative view point of ‘let's see if it settles down by itself’?*

*Spinal surgeon 1: Yes, we always do that.*

There was a concern expressed by the GPs that if spinal specialists continue to adopt this conservative ‘wait and see’ approach for some patients, this may result in spinal surgeons repeating the patient’s original MRI scan:

#### Extract 8

*GP 5: The difficulty is sometimes if the spinal surgeons see people at too early a stage they will just want to try conservative management anyway … and then repeat the scan.*

The extent to which the ‘stepped’ care model is strongly embedded within usual care for patients with short symptom durations was also reflected in how some patients understood the treatment options available to them following ‘fast-track’:

#### Extract 9

*Patient ID 8: They [the spinal physiotherapist] said ‘well this is the course of how we treat sciatica’. You wouldn’t go for the big drug [an analogy for the most intensive treatment] as a first option, you would go for the small one to see if it had any effect. The big drug would be the last option, because if that doesn’t work, where do you go from there? (Male, aged 42)*

### Perceived benefits of the ‘fast track’ care pathway

Both patients and all three clinician groups perceived benefits from the ‘fast-track’ pathway in providing earlier patient reassurance based on MRI scan findings, particularly in enabling patients to understand the cause of their pain and assuring them that there was no serious underlying pathology:

#### Extract 10

*Patient ID 6: I was happy when I had the MRI because I knew what it was [causing the pain]; I think that’s half the problem, because you worry about it otherwise. You think ‘oh my god, what’s going on there?’ But he [the spinal specialist physiotherapist] showed me the MRI scan and showed me exactly where the disc bulge was so at least then you know exactly what was going on … I know what I’m coping with and I just feel easier now. That’s 90% of the battle really, that I know that it’s nothing too sinister. (Female, aged 60)*

#### Extract 11

*Spinal physiotherapist 1: With a lot of these patients it’s the first episodes of these types of problems that they’ve ever had and they can be quite dramatic. So there is a huge amount of anxiety … and one of the benefits of ‘fast-tracking’ them is at least we can show them, ‘yes, there is something’, because if they’ve had a scan that confirms the changes … there’s nothing sinister or nasty going on. So you can offer a level of reassurance.*

Several patients also highlighted that the ‘fast-track’ pathway led to a greater sense of satisfaction with the care they had received when compared to previous experiences of NHS care. Notably, four patients reported satisfaction with the ‘fast-track’ pathway despite not having experienced any improvement in their sciatica symptoms at the point of their interview. All four had a history of episodic sciatica, and reported satisfaction in feeling that something active was being done to address their pain, which to them represented progress compared with previous care received:

#### Extract 12

*Patient ID 4: I feel like I’ve got further with you this time than I’ve got with anyone else. In previous years I’ve been to so many people for help and everyone seems to close the door on me, basically, that’s how I feel. This time it just seems like I’m getting somewhere. (Female, aged 49)*

There was some variation within the clinician data about the potential of the ‘fast-track’ pathway to benefit patient outcomes over time. One spinal surgeon felt that the main benefits would be on short-term patient outcomes, e.g. getting patients back to work sooner, and that there may be less impact in the longer-term:

#### Extract 13

*Spinal surgeon 2: If the question is: a year down the line will they be better off if they’d been treated sooner rather than treated later, the answer is probably no. The long term outcome varies very little according to the speed at which they’re treated; but in terms of getting them back to work, the sooner we treat them, the sooner we will get them back to that happy situation.*

However, 4 GPs highlighted the potential for longer-term benefits of ‘fast-tracking’ in terms of intervening earlier in order to prevent patients from developing a chronic pain problem and becoming dependent on medication:

#### Extract 14

*GP 8: I think it’s preventing people from going down into that chronicity and all the other issues that go alongside that, with dependency, like Diazepam, health-seeking … the behaviour around being an invalid.*

### Waiting times for onward treatment following ‘fast-track’

The final theme relates to patients’ and clinicians’ views on waiting times for treatment following assessment at the spinal interface clinics and onward referral. Although patient management following ‘fast-track’ was not altered as part of the trial (i.e. patients referred for onward treatments joined usual NHS waiting lists), understanding views on the ‘fast-track’ pathway within the context of the broader care pathway for patients with sciatica is an important part of understanding its overall acceptability.

Patients generally reported feeling satisfied with decisions about onward referrals following assessment at the spinal interface clinics. However, they highlighted the significant difference between the short timeframe for the initial ‘fast-track’ to MRI scan and spinal specialist opinion (on average 11.5 days between randomisation and spinal specialist appointment across the 20 patients) and the usual NHS waiting times after onward referral for more invasive treatments. For instance, the average waiting time for the four participants who received an epidural injection was 49.6 days (i.e. seven weeks) from the time of referral, and the average waiting time for patients who were referred straight for an appointment with a spinal surgeon was 57 days (i.e. 8 weeks). Interview participants who joined these usual NHS waiting lists reported uncertainty about how long they would be waiting to receive further treatments, leading to dissatisfaction:

#### Extract 15

*Interviewer: And how quickly did you go from the clinic to the MRI?*

*Patient ID 9: Very quickly, that was a matter of weeks. But then waiting for the injection they said would be about a five week waiting list... So five weeks came, and six weeks came, which kept going on and on. And I was phoning them and I was still seeing my own doctor, and he was like “We need to get something sorted with this.” And I was phoning and phoning them and an appointment then came through as a cancellation … So after initially saying, it’s sort of about a five week wait, it was longer and it would have been even longer still. (Female, aged 44)*

For four patients, the speed of their initial ‘fast-track’ coupled with what they perceived as the thorough nature of the investigation led them to believe their condition must be severe in order to warrant such prompt attention. Thus, having been ‘fast-tracked’ for MRI and spinal specialist assessment, they expected they would similarly receive any required further treatment quickly; that is, despite the patient information about the trial informing them that this would not be the case. This suggests that whilst MRI scan findings can provide reassurance, as highlighted earlier, for some patients being ‘fast-tracked’ had the opposite effect, leading them to believe they had a particularly serious problem. As a result, joining usual NHS waiting lists for further treatment led to a degree of frustration and distress:

#### Extract 16

*Patient ID 13: Because of the speed I went from the doctor to there [the SCOPiC clinic], to the MRI to the specialist; I thought ‘yeah there’s got to be something wrong here’, for them to have spent the 9 hours and so on, on treatment to get me to this point. And then for it to stop, you think, ‘well where do I go from here? Who can I talk to just to get things moving?’ (Female, aged 45)*

Following ‘fast-track’ 4 patients received a physiotherapy referral at the same time as being referred for a caudal epidural injection (see Fig. 1, earlier). The explanation patients gave about this decision was that due to the anticipated waiting time for epidural injections, they had been referred for physiotherapy to see if this might improve their symptoms whilst they wait for the injection:

#### Extract 17

*Patient ID 10: The physio said, “Right the next step is physio and we'll wait for the injection”. We'll do that while we wait for that one [i.e. the epidural injection]*

*Interviewer: So they said have the physio while you're waiting?*

*Patient ID 10: While we're waiting, yeah.*

*Interviewer: To see if that can help in the meantime?*

*Patient ID 10: Yes, that’s right. (Male, aged 57)*

However, not all patients expressed dissatisfaction with waiting times for onward treatments. In fact, some patients had been offered invasive treatments but decided against these following discussions with the clinician. These decisions were often informed by patients weighing up the perceived risks and benefits associated with different treatment options:

#### Extract 18

*Patient ID 5*: *I was offered the steroid treatment [i.e. epidural steroid injection] by the physio. I didn’t really want to take it on because she said there were risks involved; because I said at the moment I can tolerate it, it’s not that bad. If it got to the stage where it was crippling and I couldn’t get out of bed and stuff and couldn’t dress myself, I’d have to think about having something done. That would forfeit the risk involved then. (Male, aged 44)*

Clinicians across all three groups also expressed concerns about patients having to go onto usual NHS waiting lists to receive further treatments following ‘fast-track’. Echoing the views of patients in Extracts 15 and 16 above, four clinicians (2 surgeons, 2 GPs) felt that ‘fast-tracking’ patients for an MRI scan and spinal specialist consultation could result in patients anticipating that their sciatica symptoms will be resolved quickly, leading to unmet expectations if they then have to wait several weeks or months for further treatments:

#### Extract 19

*Spinal surgeon 3: There’s no point in investigating these patients quickly if you then have to put them on a long waiting list before they’re allowed to have them [i.e. spinal injections]. It’s unfair to get patients’ expectations up.*

It is important to note that, as highlighted earlier in relation to the first theme, patients would still receive further treatments sooner overall as result of getting onto NHS waiting lists sooner; but nevertheless, not being able to influence the timing of the receipt of further treatments once they joined the waiting lists was seen as a limitation of the ‘fast-track’ pathway:

#### Extract 20

*Spinal physiotherapist 4: We can ‘fast-track’ it to a point but then we can’t then necessarily influence the speed with which the next step, the next intervention occurs. So if we do deem that person as being appropriate for having an injection, but then there’s a long waiting time for injections, that’s out of our hands. So whilst we can get them to this point [i.e. MRI scan and spinal specialist opinion] quicker we can’t then necessarily get them onto the next stage any quicker.*

## Discussion

### Exploring the identified themes in relation to normalisation process theory (NPT) and the ‘boundary object’ concept

The findings presented indicate that all groups perceived benefit from the ‘fast-track’ care pathway to MRI scan and spinal specialist consultation in the stratified care approach tested in the SCOPiC trial; both in relation to clinical outcomes and in providing reassurance to patients based on MRI scans. When explored through the lens of NPT, these findings suggest that the ‘fast-track’ pathway was able to establish a degree of ‘coherence’ (i.e. made sense) to both patients and clinicians, in that they appeared to ‘buy in’ to the aims of the ‘fast-track’ pathway, and saw it as making a meaningful improvement to current care. Considered in relation to Allen’s [22] conceptualisation of care pathways as ‘boundary objects’, the ‘fast-track’ pathway can be seen to have, at least in part, enabled the bringing together of the different stakeholders’ goals, particularly in relation to reassuring patients with sciatica early on.

This reassurance related to patients receiving an explanation for the cause of their pain and feeling assured that there was no serious underlying pathology; what Pincus et al. [28] classify as ‘cognitive reassurance’. This finding contrasts with other literature, including arguments by Wheeler et al. [29], that imaging patients with LBP can lead to distress if aberrant findings are identified. The reason for the difference in findings may be that in the case of non-specific LBP, often no obvious cause of pain is identified through scan results, leading patients to experience frustration and uncertainty towards unexplained symptoms [30]. Or, as Wheeler et al. suggest, imaging may show up ‘abnormalities’, which ‘frequently fall within the range of age-related norms’, but which patients perceive as indicating ‘damage’, causing them to feel anxious rather than reassured. However, in the case of sciatica, a clear biomedical cause is more often present, as was the case with all the patients interviewed in this study who received an MRI scan. This meant that patients better understood their condition and had more certainty surrounding symptoms, leading to stronger cognitive reassurance. It could be suggested that understanding the cause of their pain was particularly important for this group of individuals with sciatica, as part of the reason for many of them having been stratified to the ‘fast-track’ subgroup was that they initially experienced acute, severe pain.

It may be that the explanation of the MRI scan results also contributed to this cognitive reassurance, as was shown in Extract 10, earlier, in which the patient highlighted the explanation she was given by the clinician about the imaging results as contributing to her reassurance. Ong et al. [31] have similarly highlighted the importance patients with sciatica place on receiving clear information about their diagnosis from clinicians. On the SCOPiC fast-track pathway, MRI scan results were communicated by a spinal specialist clinician (physiotherapist), who, given their knowledge and experience, are in a position to communicate results in a clear and expert manner. This finding could indicate that who (i.e. which professional) delivers the scan results, and how these are communicated to the patient can have important effects on the patient’s level of reassurance.

However, not all patients reported feeling reassured, and for four patients the speed of receiving the scan and seeing the spinal specialist led to concerns that their condition was particularly severe, which in contrast to the above suggests a lack of coherence of the ‘fast-track’ pathway. This highlights the importance of clinicians giving a clear explanation to patients about why they are being referred for investigation and spinal specialist opinion, so that this does not result in unnecessary worry on the part of patients.

Despite this, all patients reported being pleased with the short timeframe for receiving an MRI scan and spinal specialist consultation, similar to the previous findings about the importance patients with sciatica place on early investigation [32]. However, this may represent a mismatch between the respective goals/agendas of the patients and clinicians that is highlighted in the ‘boundary object’ concept. Whilst patients may perceive waiting times for investigations from a mind-set of ‘the sooner the better’, some clinicians raised concerns about the suitability of patients with acute symptoms for early investigations, and a reticence about early MRI scans. For these clinicians, the ‘fast-track’ pathway appeared less successful in achieving ‘coherence’, as there was a concern that ‘acute’ patients’ symptoms may well resolve without the need for imaging and spinal specialist consultation. Some spinal surgeons and GPs did feel it was acceptable for patients with short symptom duration to be ‘fast-tracked’, but only to provide early reassurance rather than to direct treatment.

In general, there was a reluctance among clinicians to consider more invasive treatments following ‘fast-track’ for patients with symptom durations of less than 6 weeks, or even 2–3 months. This appeared to reflect the overall expectation of a favourable natural course of most patients with sciatica, as well showing the degree to which the current ‘stepped’ care approach is strongly embedded in routine clinical care; clinicians were reticent to move away from this approach. Intervening too early with these patients therefore represented a lack of ‘coherence’ with usual ways of working. These views show similarity with findings in other healthcare contexts; Hofstede et al. [33] observed in the Dutch context a reluctance on the part of clinicians to refer patients with short symptom duration for investigations and specialist opinion before a period of conservative management has first been tried, and failed. In the context of the SCOPiC trial, this meant that for some patients, having initially been ‘fast-tracked’ they then received conservative treatments similar to those received by patients who were not on the fast-track pathway. This was reflected in Fig. 1, earlier, which shows that 16 of the 20 patients interviewed were referred for physiotherapy as a first treatment option following their spinal specialist assessment.

A lack of coherence of the ‘fast-track’ pathway was also evident in views towards broader organisational/system level factors [16], in that both patients and clinicians highlighted limitations in the ‘fast-track’ pathway in not being able to influence waiting times for further treatment. Some clinicians did show a recognition that ‘fast-tracking’ patients through the initial phase of the care pathway results in them waiting less time overall for treatments when compared to usual care. However, clinicians and patients highlighted the incongruence between the short ‘fast-track’ timeframe for patients receiving an MRI scan and spinal specialist opinion (on average 11.5 days), compared with the usual NHS waiting times for receiving further treatments such as epidural injections (on average 7 weeks for the four interview participants who received an epidural injection during the trial). Particularly for patients, the ‘fast-track’ pathway was seen as less ‘meaningful’ given that ‘fast-track’ did not extend to the receipt of these further treatments.

### Contribution of findings in providing insights on the ‘fast-track’ pathway tested in the RCT

Patients in the stratified care arm of the trial showed a small but not statistically significant difference in time to first resolution of symptoms (those managed using stratified care reached resolution 2 weeks earlier than those in non-stratified care; a median of 10 weeks versus 12 weeks) [10]. Similarly, exploratory, pre-specified subgroup analysis showed a small difference of 1 week (median) in time to resolution of symptoms, in favour of stratified care for the ‘fast-track’ patient group. The findings presented in this paper can provide useful insights for understanding the fast-track pathway and the outcomes of patients on it. Of the 20 patients interviewed, following spinal specialist consultation 16 were referred for conservative treatment, and four were referred to a spinal surgeon (see Fig. 1, earlier). This could be in part down to the lack of ‘coherence’ clinicians indicated in considering invasive treatment options for patients with short symptom duration, and is indicative of the overall preference of clinicians to allow nature some time to take its course in sciatica, before more invasive treatments are used. However, even when invasive treatments were recommended by spinal specialists, some patients reported instead choosing to opt for physiotherapy. Therefore, whilst the findings showed that both patients and clinicians reported benefits of the ‘fast-track’ pathway, there was collective clinical reluctance, and reluctance from some patients, to consider invasive treatments early on. In this respect, therefore, this facet of the stratified care model tested in the trial was not consistently helpful to clinicians in their discussions with their patients about management options.

Additionally, whilst the aim of the ‘fast-track’ pathway was only to shorten the timeframe for patients to receive an MRI scan and spinal specialist consultation – which was successfully achieved for those patients interviewed – patients and clinicians felt that ‘fast-track’ did not extend far enough, they wanted the ‘fast-tracking’ applied to the whole patient pathway (i.e. from first presentation in general practice, through to the receipt of invasive treatments, if required), rather than joining usual NHS waiting lists for further treatment.

### Strengths and limitations

A strength of this study is the parallel investigation of both patients’ and clinicians’ views, allowing access to a range of different perspectives about the ‘fast-track’ pathway that was tested in the SCOPiC trial. The total sample of 40 interview participants was suitably large to allow for the identification of trends across the dataset, and the use of the two theoretical frameworks – NPT and the ‘boundary objects’ concept – enabled us to develop a more robust understanding of the identified issues. The multidisciplinary team involved in data analysis was a further strength; as well as PPIE input into the interpretation of the patient data, which increases the trustworthiness of the findings presented.

A potential limitation is that interviews with spinal surgeons and GPs commonly involved hypothetical discussions about patients in the ‘fast-track’ pathway, rather than reflecting on concrete experiences of specific patients in the SCOPiC trial (any one GP or surgeon only saw small numbers of patients in the trial spread over a two-year period, thus hampering recall). This compared with spinal physiotherapists who were able to draw on examples of patients they had seen in spinal interface clinics through the ‘fast-track’ pathway. However, the adoption of the ‘fast-track’ pathway requires communication between clinicians across the care pathway and therefore gaining the views of these different clinician groups as to the acceptability of this approach holds important insights.

The study is in some ways particular to the UK NHS setting, which could limit the applicability of findings to other healthcare contexts. For instance, whilst physiotherapists can develop into roles as spinal specialist clinicians in many healthcare systems other than the UK ─ and therefore this role is not entirely unique to the UK NHS ─ we acknowledge that this may be unusual in some countries. Additionally, the issues we have identified in relation to waiting times following referral to secondary care services is different in the NHS when compared to many other countries, particularly those in which healthcare is accessed through private healthcare insurance. However, despite these differences between healthcare systems, several of the findings presented do have broader applicability across different healthcare contexts. In particular, views about the role of early imaging and spinal specialist opinion in providing reassurance to patients with severe sciatica symptoms, and the collective clinical reluctance observed towards moving away from a stepped care approach in managing sciatica of short duration, with a preference for early conservative management to allow for the possibility of natural resolution of symptoms, have relevance to clinical practice beyond the UK setting.

When interpreting these findings, it is also important to acknowledge the influence of the researcher’s contributions on participants’ interview responses. The interviewer was part of the SCOPiC trial team, and therefore his close involvement with the trial could have had the potential to influence the way in which participants’ views were elicited. It was made explicit to participants that the interviewer was part of the trial team; however, it was also emphasised to participants that we were interested in investigating both positive and negative aspects of participants’ experiences, and the variation in views observed suggests that participants were not led into adopting a particular stance in line with that of the interviewer.

## Conclusion

This paper explored patients’ and clinicians’ perceptions of a ‘fast-track’ pathway to MRI scan and spinal specialist consultation for patients with sciatica consulting in primary care, offered as part of a new stratified care model tested within the SCOPiC trial. Whilst the ‘fast-track’ pathway was found to achieve a degree of ‘coherence’ [16] in that both patients and clinicians perceived added value, particularly in providing early reassurance, the pathway was less ‘meaningful’ to some clinicians for managing patients with acute symptoms (typically less than 6 weeks). This indicated a general reluctance on the part of clinicians to intervene too early, reflecting the expectation of natural resolution in most cases of sciatica, as well as a reticence to move away from the current, ‘stepped care’ model. Both patients and clinicians also felt that the ‘fast-track’ pathway was limited in that it did not extend to shorten the time patients waited for further treatments after their spinal specialist consultation; therefore, whilst the principle of ‘fast-tracking’ patients was seen as acceptable, it was perceived as being limited in scope.

These findings help us to better understand this aspect of the stratified care intervention tested in the trial. Based on the RCT findings outlined earlier (see [10] for further details), there is no strong justification to implement this model of stratified care for patients with sciatica, but other models of stratification could be developed and would require testing. With this in mind, the qualitative findings presented here have implications for future intervention design, as well as future management of patients with sciatica. Firstly, future research exploring early referral of patients with sciatica with severe symptoms for consideration of treatments such as surgery or spinal epidural injections must account for and address the difficulties in bringing about clinician behaviour change, given that the current stepped care approach was found to be so strongly embedded in routine practice for the care of patients with short duration of symptoms. Secondly, findings also indicate the importance that both clinicians and patients place on ‘cognitive reassurance’ [28] being given to patients with severe sciatica symptoms, and the role that imaging can play in providing this type of reassurance in cases where imaging reveals a clear explanation for the patient’s pain, and where this is accompanied by a thorough explanation from a trusted clinical expert.

## Supplementary information

**Additional file 1 Appendix.** Interview Topic Guide: Patients

## Data Availability

In line with the Standard Operating Procedures in place at the School of Primary, Community and Social Care, where this study was conducted, data is archived at a dedicated location within the Keele CTU network. A request to access archived data can be made by completion of a Data Transfer Request form, which can be accessed by contacting the school directly: School of Primary, Community and Social Care, Keele University, Staffordshire, ST5 5BG; Tel: + 44 (0) 1782 733905.

## Abbreviations

SCOPiC: SCiatica Outcomes in Primary Care
GP: General Practitioner
RCT: Randomised Controlled Trial
NHS: National Health Service
NPT: Normalisation Process Theory
