# Supplementary material for: Patients’ and clinicians’ perspectives on a ‘fast-track’ pathway for patients with sciatica in primary care: qualitative findings from the SCOPiC stratified care trial
Source: BMC Musculoskelet Disord. 2020 Jul 17;21:469. doi: 10.1186/s12891-020-03483-z (PMC7367249; doi:10.1186/s12891-020-03483-z)
Supplement: Supplementary file 1 — Additional file 1 Appendix. Interview Topic Guide: Patients [file 12891_2020_3483_MOESM1_ESM.docx]

The SCOPiC trial

Interview Topic Guide: Patients

1. **Introduction**

Check that participant has read and understood the PIS – invite questions.

Explain arrangements for: consent, recording, anonymity etc.

1. **Experience of sciatica** (where possible invite participants to expand on their responses)

Can you tell me about your experiences of sciatica? probe for symptoms, duration, effects on activities of daily living, work if applicable, relationships, mood, etc.

1. **Experience and acceptability of fast-track**
2. Please tell me what first brought you to visit your GP and what happened from there?
3. What were your experiences of the care you received?
4. Did the care you received meet your expectations – if yes, can you elaborate on this? If not, how did it differ to what you were expecting?
5. How satisfied do you feel with the care you received, and why? Prompts around timeliness, appropriateness of clinician seen and treatment received.
6. How have your sciatica symptoms been since you started receiving this treatment? – Have they been resolved? To what extent? How satisfied with outcome?
7. **Close of discussion**
8. Summary of discussion: any additional remarks?
9. Check consent is still in place.
10. Check if participant would like to receive a summary of the interview findings.

The SCOPiC trial

Interview Topic Guide: GPs

1. **Introduction**

Check that participant has read and understood the PIS

Explain arrangements for: consent, recording, anonymity, expenses where appropriate etc.

1. **Acceptability of fast-track referral for high risk patients:** (where possible invite participants to expand on their responses)
   1. What are your views about fast-tracking sciatica patients for specialist spinal assessment?
   2. How does this compare with your usual practice?
   3. In general, do you feel that the fast-track pathway was beneficial to patients in the SCOPiC study? – if yes, then how? If no – why do you feel it was not beneficial?
   4. Which patients might benefit most from this fast-track pathway?
   5. Speaking generally, were there fast-tracked patients who you feel did not benefit from a clinical perspective? – prompt participant to elaborate on response.
   6. Are there some patients for whom a fast-track pathway would not be appropriate? If yes, why?
   7. Based on your experiences of the SCOPiC study, how acceptable do you consider this approach to be in treating patients with severe sciatica symptoms?
   8. How confident do you feel in using this fast-track approach in the future?
   9. How might this approach affect workload or relationships with other healthcare professionals in the care pathway?
   10. What in your opinion might be the added value of fast-track referral compared to usual care, for sciatica and suspected sciatica patients?
   11. What, if any, drawbacks might there be?
   12. Are there resource issues that need to be considered? If so, what?
   13. What additional training/skills might be needed?
2. **Close of discussion**
   1. Summary of discussion: any additional remarks?
   2. Check consent is still in place.
   3. Check if participant would like to receive a summary of the interview findings.

The SCOPiC trial

Interview Topic Guide: Spinal Physiotherapists

1. **Introduction**

- Check that participant has read and understood the PIS
- Explain arrangements for: consent, recording, anonymity, expenses where appropriate etc.
- Ask for a bit of information about participant, e.g. specialist interest, length of time in practice etc.

1. **Acceptability of fast-track referral for high risk patients:** (where possible invite participants to expand on their responses)
   1. What are your views about fast-tracking sciatica patients for specialist spinal assessment?
   2. How does this compare with usual practice from your perspective in terms of pathway for sciatica patients?
   3. Is there a difference in the way you manage these fast-track patients in the interface clinic?
      1. E.g. does it make a difference knowing that a patient has been stratified as being suitable for fast-track?
      2. Do you feel confident in your role within this pathway in giving specialist opinion and in the use of this approach in the future?
   4. In general, do you feel that the fast-track pathway was beneficial to patients in the SCOPiC study? – if yes, then how? If no – why do you feel it was not beneficial?
   5. Which patients might benefit most from this fast-track pathway?
   6. Speaking generally, were there fast-tracked patients who you feel did not benefit from a clinical perspective? – prompt participant to elaborate on response.
   7. Are there some patients for whom a fast-track pathway would not be appropriate? If yes, why?
   8. Based on your experiences of the SCOPiC study, how acceptable do you consider this approach to be in treating patients in the fast-track group?
   9. How might this approach affect workload or relationships with other healthcare professionals in the care pathway? E.g. GPs, surgeons.
   10. What in your opinion might be the added value of fast-track referral compared to usual care, for sciatica and suspected sciatica patients?
   11. What, if any, drawbacks might there be?
   12. Are there resource issues that need to be considered? If so, what?
   13. What additional training/skills might be needed?
2. **Close of discussion**
   1. Summary of discussion: any additional remarks?
   2. Check consent is still in place.
   3. Check if participant would like to receive a summary of the interview findings.

The SCOPiC trial

Interview Topic Guide: Spinal surgeons

1. **Introduction**

- Check that participant has read and understood the PIS
- Explain arrangements for: consent, recording, anonymity, expenses where appropriate etc.
- Record key info about participant: background, specialist interest, length of time in practice etc.

1. **Acceptability of fast-track referral for sciatica patients:** (where possible invite participants to expand on their responses)
   1. What is the usual process through which sciatica patients are referred onto you for surgical opinion? – do you see any differences resulting from this fast-track approach?
   2. In your view, what sorts of characteristics make a patient with sciatica suitable for surgical opinion?
   3. What are your views about fast-tracking sciatica patients for specialist spinal assessment (including MRI)?
      1. Prompt around timeframe issues/ appropriateness of seeing patients at an earlier stage etc.
   4. Do you feel that fast-tracking sciatica patients who have high probability of no improvement and are likely to be referred for a specialist opinion at some point anyway, could make a difference to your practice?
      1. E.g. The number or types of sciatica patients being referred to you? - could there be greater/ lesser number of referrals? Seeing patients at an earlier stage etc.
   5. In general, do you feel that the fast-track pathway may be beneficial to patients in the SCOPiC study? – if yes, then how? If no – why do you feel it would not be beneficial?
   6. Which patients might benefit most from this fast-track pathway?
   7. Are there some patients for whom a fast-track pathway would not be appropriate? If yes, why?
   8. How acceptable do you consider this approach to be in assessing patients?
   9. What, if any, drawbacks might there be?
2. **Close of discussion**
   1. Summary of discussion: any additional remarks?
   2. Check consent is still in place.

Check if participant would like to receive a summary of the interview findings
